# Reliable pain and function outcomes but limited sport performance after high tibial osteotomy for medial knee osteoarthritis in the grey zone between osteotomy and unicompartmental replacement

**DOI:** 10.1002/ksa.70223

**Published:** 2025-12-08

**Authors:** Tomas Pineda, Antoine Piercecchi, Christophe Jacquet, Nicolás Gaggero, Kristian Kley, Matthieu Ollivier

**Affiliations:** ^1^ Facultad de Medicina, Hospital del Trabajador Universidad Andrés Bello Santiago Chile; ^2^ Facultad de Medicina, Hospital el Carmen Universidad Finis Terrae Santiago Chile; ^3^ Department of Biomechanics, APHM, CNRS, ISM, St. Marguerite Hospital, Institute for Locomotion Aix‐Marseille University Marseille France; ^4^ Department of Orthopedic Surgery, Institute for Locomotion Aix‐Marseille University Marseille France; ^5^ Orthoprofis Hannover Germany; ^6^ Orthopaedic Innovation London UK

**Keywords:** AKUMA framework, function, high tibial osteotomy, medial compartment osteoarthritis, pain, return to sports

## Abstract

**Purpose:**

To evaluate mid‐term outcomes of high tibial osteotomy (HTO) in patients with medial knee osteoarthritis presenting borderline indication between osteotomy and unicompartmental knee arthroplasty (UKA) and to identify clinical and radiographic factors associated with success in pain, function and sport.

**Methods:**

Retrospective multicentre cohort of consecutive HTOs performed between 2005 and 2015 with ≥2 years of follow‐up, including patients classified within the grey zone according to the AKUMA framework. Preoperative and postoperative long‐leg radiographs were obtained to measure hip–knee–ankle (HKA) angle, mechanical lateral distal femoral angle (LDFA), medial proximal tibial angle (MPTA) and joint line convergence angle (JLCA). Primary outcomes were Western Ontario and McMaster Universities Osteoarthritis Index (WOMAC), Tegner and modified Weiss (mW) scores; secondary outcomes included satisfaction, sports relevance, symptom‐free return to sport and forgotten‐knee. Success thresholds were WOMAC ≥ 80, Tegner ≥5 and mW ≥6. Univariate analyses compared successes versus non‐successes, and multivariable logistic regression was performed for WOMAC.

**Results:**

Eighty‐four patients were analysed with a mean follow‐up 8.0 ± 3.2 (2.0–14.3) years. Success rates were 72.0% for WOMAC, 6.8% for Tegner and 7.5% for mW. Postoperative MPTA was higher in WOMAC successes than in non‐successes. In multivariable analysis, Ahlbäck Grade 3 lowered the odds of achieving WOMAC ≥ 80 (odds ratio [OR]: 0.20, 95% confidence interval [CI]: 0.04–0.94). Tegner success was associated with greater LDFA and with rating sport as very important. mW success was associated with lower preoperative MPTA, lower LDFA, higher tibial extra‐articular deformity (TEAD) and very high sport priority. Higher Ahlbäck and Kellgren–Lawrence (KL) grades were linked to worse satisfaction; the forgotten‐knee endpoint showed no significant associations.

**Conclusion:**

In medial OA knees within the AKUMA grey zone, HTO provides reliable pain relief and functional improvement, while sport success remains limited. Osteoarthritis burden reduces the probability of high pain and function‐related scores, while sport‐oriented outcomes depend more on coronal alignment features and the patient′s sport priority.

**Level of Evidence:**

Level IV.

**Abbreviations**
BMI: body mass index
EAD%: extra‐articular deformity contribution percentages
FEAD%: femoral extra‐articular deformity percentages
HKA: hip–knee–ankle
HTO: high tibial osteotomy
IAD%: intra‐articular deformity contribution percentages
JLCA: joint line congruency angle
KL: Kellgren–Lawrence
mLDFA: mechanical lateral distal femoral angle
MPTA: medial proximal tibial angle
mW: modified Weiss score
OA: osteoarthritis
SD: standard deviation
TEAD%: tibial extra‐articular percentages
UKA: unicompartmental knee arthroplasty

## INTRODUCTION

Young, active patients with medial knee osteoarthritis (OA) present a persistent challenge in surgical decision‐making. The choice between high tibial osteotomy (HTO) and unicompartmental knee arthroplasty (UKA) goes far beyond age or activity level, relying on a complex interplay of coronal alignment, the location and extent of joint damage, soft‐tissue balance and individual patient priorities [5, 6, 15, 16, 19, 21].

HTO and UKA are conceptually different procedures for medial compartment OA. HTO corrects extraarticular varus deformity and redistributes load across the joint, whereas UKA addresses focal intraarticular cartilage loss and restores local congruency. In clinical practice, however, a small subset of patients presents overlapping features of both deformities, in whom either option may be reasonable depending on individual anatomy and goals. This group has not been clearly characterized in the literature, highlighting the need for structured frameworks capable of defining these borderline cases.

The recently introduced AKUMA framework was developed to bring structure to this complexity as a conceptual tool to characterize deformity distribution and OA severity [15].

By integrating overall coronal alignment, the distribution of deformity along the limb and OA severity, it stratifies patients into three categories: those more suitable for osteotomy, those more suitable for UKA and a grey‐zone group with overlapping features in whom either option may be appropriate.

However, robust evidence focusing specifically on grey‐zone knees remains scarce. Surgeons continue to lack outcome‐driven guidance to determine which patients derive the greatest benefit from HTO and how to set realistic expectations for pain relief, function and return to sport [5, 6, 16, 19].

This study aims to evaluate the mid‐term outcomes of HTO in AKUMA grey‐zone knees and to identify the clinical and radiographic factors associated with success across pain, function and sport domains.

## MATERIALS AND METHODS

### Study design and patient selection

We conducted a retrospective multicenter cohort study of consecutive patients who underwent HTO between 2005 and 2015. Inclusion criteria were age between 20 and 60 years at the time of surgery, varus alignment defined as a mechanical hip–knee–ankle (HKA) angle <180° and a minimum follow‐up of 2 years. Exclusion criteria included prior osteotomy or ligament surgery, bilateral cases, and, according to the AKUMA framework, classification into the Osteotomy or UKA groups. Consequently, only knees within the AKUMA grey zone were included to enable a focused evaluation of this subgroup.

### Data collection and radiographic assessment

Demographic data included age, sex, body mass index (BMI) and operated side. Standardized long‐leg standing radiographs were reviewed to record [15]:

- Mechanical lateral distal femoral angle (mLDFA): Angle between the line tangent to distal femoral condyles and the mechanical axis of the femur.
- Medial proximal tibial angle (MPTA): Angle between the line tangent to the tibial plateau and the mechanical axis of the tibia.
- Hip–knee–ankle angle (HKA, global deformity, GD): Angle between a line from the centre of the femoral head to the centre of the knee joint and the line from the centre of the knee to the centre of the ankle joint.
- Joint line congruency angle (JLCA): Angle between a line tangent to the distal femoral condyles and a line tangent to the proximal tibial plateau.
- Femoral and tibial extra‐articular deformity contribution percentages (FEAD%, TEAD%).
- Intra‐ and extra‐articular deformity contribution percentages (IAD%, EAD%).

OA severity was graded using the Ahlbäck and Kellgren–Lawrence (KL) classifications.

All radiographic measurements were performed at each centre by a fellowship‐trained knee surgeon. The derived percentage‐based parameters were subsequently calculated by a single surgeon (T. P.) who performed the overall analysis according to the AKUMA framework, ensuring consistent application of the grey‐zone classification.

### Sport participation variables

Sport‐related variables included:

- Perceived importance of sport (five‐level scale: not important to very important).
- Frequency of participation (five‐level scale: never to >2 times/week).

### Outcomes

Primary outcomes were patient‐reported Western Ontario and McMaster Universities Osteoarthritis Index (WOMAC), Tegner and modified Weiss (mW) scores [13, 22]. The WOMAC score was evaluated on a 0–100 scale, where higher values indicate less pain and better function. The mW score, adapted from the Weiss and Noble Total Knee Function Questionnaire for OA cohorts, summarizes sport‐related function by combining three components: activity frequency, patient‐perceived importance and symptom‐related bother. Higher scores indicate better function.

Success thresholds were defined as WOMAC ≥ 80, Tegner ≥5 and mW ≥6. Secondary outcomes included patient satisfaction (very satisfied/satisfied/dissatisfied/disappointed), return to sport without symptoms (yes/no) and presence of a forgotten knee (yes/no).

### Statistical analysis

Continuous variables are reported as mean ± standard deviation (SD) and categorical variables as counts (percentages). Normality was tested using the Shapiro–Wilk test. Depending on distribution, continuous variables were compared using the independent‐samples *t* test or Mann–Whitney *U* test. Categorical variables were compared using the *χ* ^2^ test or Fisher's exact test, as appropriate.

Given the limited number of events, multivariable modelling was restricted to WOMAC success, analysed using binary logistic regression. Covariates were selected a priori based on clinical relevance and univariate signals. Tegner and mW were analysed univariately, as <10 successes precluded reliable multivariable modelling. All analyses were performed using SPSS Statistics (Version 27.0.1.0; IBM Corp.). Statistical significance was set at *p* < 0.05.

## RESULTS

A total of 84 knees met eligibility criteria, with a mean follow‐up of 8.1 ± 3.2 years (range, 2.0–14.3). According to OA grading, most knees were Ahlbäck 2 and KL 3. General data on the study population are shown in Table 1.

**Table 1:** Patient characteristics.

Number of knees	84
Age at surgery (years)	52.3 ± 6.2 (29.4–59.9)
BMI (kg/m^2^)	28.9 ± 4.8 (17.1–42.4)
Sex	
Male	58 (69%)
Female	26 (31%)
Affected side	
Right	48 (57.1%)
Left	36 (42.9%)
OA grade	
Kellgren–Lawrence	
I	14 (14.1%)
II	19 (19.2%)
III	50 (50.2%)
IV	12 (12.1%)
Ahlbäck	
I	11 (11.1%)
II	52 (53.3%)
III	31 (31.3%)
Follow‐up period (years)	8 ± 3.2 (2−14.3)

*Note*: All values are presented as arithmetic means ± SD (minimum–maximum) or *n* (%).

Abbreviations: BMI, body mass index; OA, osteoarthritis; SD, standard deviation.

Table 2 summarizes the radiological preoperative deformity analysis and postoperative measurements.

**Table 2:** Axis deviations over the follow‐up period.

	Preoperative	Postoperative	Difference	*p* value
HKA (°)	172.9 ± 2.7	181.2 ± 3.9	8.2 ± 4.7	**<0.001**
MPTA (°)	85.1 ± 2.1	92.7 ± 3.6	5.8 ± 3.1	**<0.001**
mLDFA (°)	88.8 ± 1.6	88.8 ± 1.6	—	—
JLCA (°)	3.6 ± 1.1	3.1 ± 2.4	0.8 ± 2.6	**0.032**

*Note*: All values are presented as arithmetic means ± SD. Bold values are statistically significant.

Abbreviations: HKA, mechanical tibiofemoral angle; JLCA, joint line convergence angle; mLDFA, mechanical lateral distal femur angle; MPTA, mechanical medial proximal tibia angle; SD, standard deviation.

**Figure 1:**
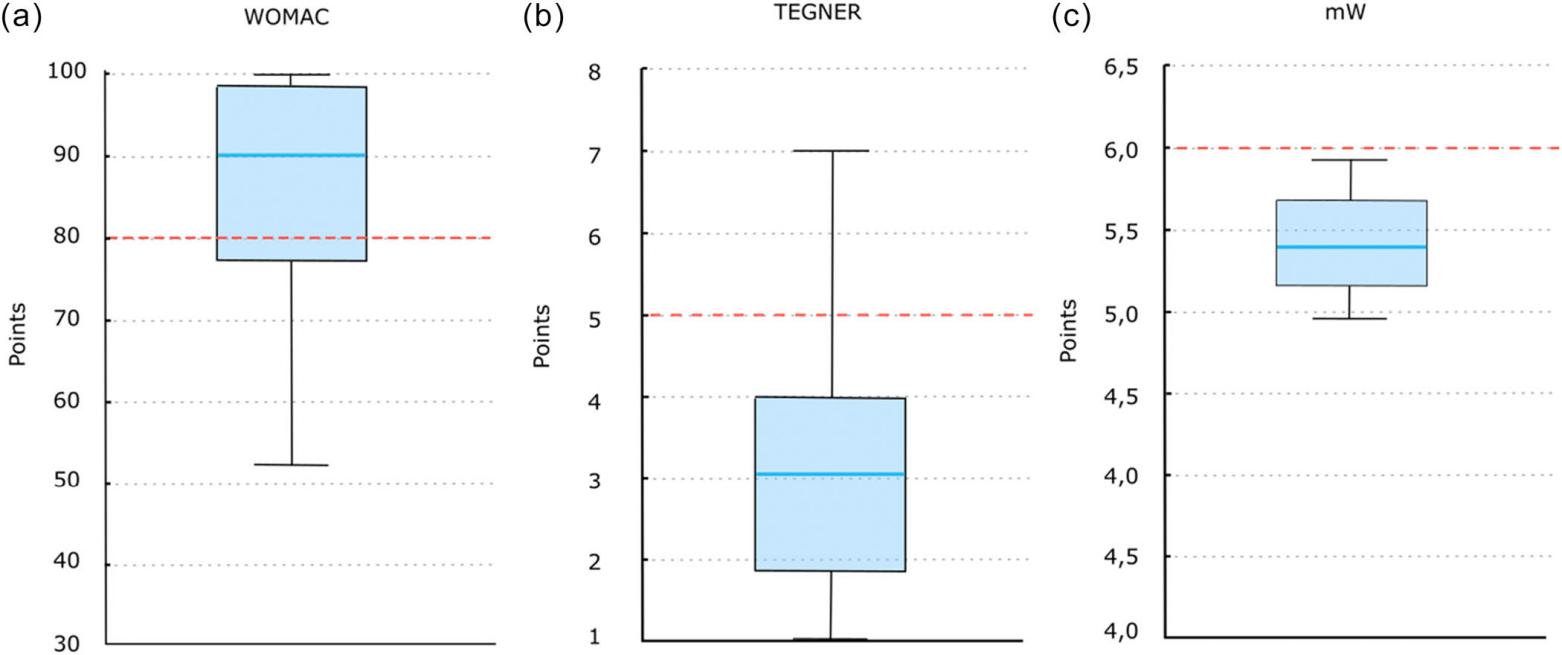
Boxplots of the primary outcomes at last follow‐up: (a) WOMAC score results, (b) Tegner score results and (c) mW Score results. The upper and lower whiskers in the representation correspond to the 97.5th and 2.5th percentiles, respectively. Red dashed lines mark the prespecified success thresholds (WOMAC = 80, Tegner = 5, mW = 6). mW, modified Weiss Score; WOMAC, Western Ontario and McMaster Universities Osteoarthritis Index.

Regarding the primary outcomes, data were available for WOMAC in 75 knees (89.2%), Tegner in 74 (88%) and mW in 68 (80.9%). The proportions achieving the prespecified success thresholds were 72.0% for WOMAC, 6.8% for Tegner and 7.5% for mW. See Figure 1.

For WOMAC success, univariate analysis identified Ahlbäck Grade 3, KL Grade 4, TEAD% ≥80, high sport relevance and a higher postoperative MPTA as significant; postoperative MPTA was greater in successes (93.3° ± 3.8) than in non‐successes (91.3° ± 3.2), mean difference +2.07° (95% confidence interval [CI]: 0.11–4.02; *p* = 0.039). In the multivariable model, only Ahlbäck Grade 3 independently predicted lower odds of achieving WOMAC ≥ 80 (odds ratio [OR]: 0.20, 95% CI: 0.04–0.94; *p* = 0.042).

LDFA was the only radiographic parameter associated with Tegner success. The success group showed a greater LDFA (90.3° ± 0.6) than the non‐success group (88.9° ± 2.1), with a mean difference of 1.35° (95% CI: 0.27–2.43; *p* = 0.022). In addition, patients who rated sport as “very important” were more likely to achieve Tegner ≥ 5 (*p* = 0.012).

For mW success, univariate analyses showed that the successful group had a lower preoperative MPTA (84.0° vs. 85.5°), a lower LDFA (87.3° vs. 88.9°) and a higher TEAD% (75% vs. 50%), with corresponding *p* values of <0.001, 0.015 and 0.035, respectively. Furthermore, rating sport as “very important” was associated with mW ≥6 (*p* = 0.015). No other demographic or radiographic measures were associated with success on any scale.

Finally, higher Ahlbäck (*p* < 0.001) and KL grades (*p* = 0.042) were associated with a less favourable satisfaction profile. Return to sport without symptoms was also associated with Ahlbäck grade (*p* = 0.032). The forgotten‐knee endpoint showed no significant associations with any of the variables assessed.

## DISCUSSION

The key finding of this study is that AKUMA grey‐zone knees treated with HTO achieved meaningful improvements in pain and daily function, but achieving higher sport‐level recovery remains uncommon in this population. At a mean follow‐up of 8 years, 72% of patients reached the predefined WOMAC threshold, yet only a minority achieved Tegner or mW success. These findings indicate that HTO can provide satisfactory pain relief and functional recovery in this challenging subgroup, although high‐level sport participation should not be expected. Recognizing this distinction is essential for surgical decision‐making and for setting realistic postoperative expectations.

To facilitate the interpretation of clinical outcomes, predefined success thresholds were established for each score based on available literature and clinical relevance. For the WOMAC score (higher = better), previous studies have shown that total scores above 75–83 are associated with a satisfactory symptom state after knee arthroplasty [8, 20]. Therefore, the WOMAC ≥ 80 threshold used in this study aligns with values reported in the literature as representing acceptable pain and function levels. Regarding sports activity, long‐term data indicate that postoperative Tegner scores of 3–4 are typical after UKA, whereas higher scores ( ≥ 5) correspond to regular, moderate sports participation and are considered a favourable but less frequent outcome [12, 20].

The mW score provides a more detailed assessment of sports‐related function, encompassing three domains—frequency of participation, perceived importance of sport and symptom‐related limitations—across multiple sporting disciplines. However, this score has been less frequently used in the literature and no validated cut‐off exists to define good or excellent results [13, 22]. Consequently, the ≥6 success threshold adopted in this study should be regarded as an arbitrary but pragmatic definition based on the scale's structure and clinical interpretability.

Our findings suggest that Ahlbäck Grade 3 independently reduced the likelihood of WOMAC success, whereas sport‐oriented outcomes were more closely associated with coronal alignment features and the importance patients attributed to sport. The boxplots illustrate this contrast, showing consistent functional improvement (WOMAC) but greater variability in sport‐related outcomes, reflecting the different determinants of success within this group of patients.

These findings align with contemporary comparative cohorts, suggesting that, with appropriate selection, opening‐wedge HTO can achieve short‐term PROMs comparable to UKA in demanding patients [5, 16]. Our results confirm that HTO provides reliable pain relief and functional improvement as measured by the WOMAC score, but also highlight that advanced medial degeneration limits the magnitude of achievable benefit. This observation reinforces the importance of grading disease severity when counselling grey‐zone candidates. Prior reports have shown encouraging survivorship and function after HTO even in advanced OA, while others identified severe chondral wear and higher radiographic grades as predictors of inferior outcomes or dissatisfaction [3, 7, 10, 14, 18, 19]. Our data are consistent with the latter.

The sport‐related results and the observed association with anatomical factors may indicate that coronal alignment and joint‐line orientation play a role in sport recovery and that tibial‐only correction may not always optimize joint‐line orientation or load‐sharing for athletic demands [1]. This echoes modern planning principles that emphasize preservation of the physiologic joint line and the distribution of correction between the femur and tibia [2, 9, 17]. In cases of femoral varus or when large tibial corrections risk excessive joint‐line obliquity, distal femoral or double‐level osteotomy may better maintain coronal mechanics [1, 2, 4, 9, 11, 17].

### Limitations

This study has several limitations. Its retrospective multicentre design and absence of a comparator cohort limit causal inference and external benchmarking. However, focusing exclusively on the AKUMA grey‐zone subgroup provides valuable information on a population that has rarely been analysed as an independent entity. Because of the study′s retrospective and multicentre nature, surgical technique, correction target and fixation method were not standardized and were left to the discretion of the operating surgeon. All procedures were tibial osteotomies, so results may not generalize to grey‐zone patients requiring femoral or double‐level corrections. Sport endpoints had low event counts, precluding robust multivariable modelling and raising the risk of type II error. Although prespecified, the success thresholds for WOMAC, Tegner and mW are somewhat arbitrary and may affect absolute rates.

## CONCLUSION

In AKUMA grey‐zone knees, HTO provides reliable mid‐term pain relief and functional gains, but success at higher levels of sport remains uncommon. The burden of OA, particularly Ahlbäck Grade 3, reduces the likelihood of WOMAC success, whereas sport‐related outcomes depend more on coronal alignment features and patient priorities. These results support HTO as a valuable option in the grey zone and emphasize the importance of planning strategies that preserve a physiologic joint line for patients aiming to return to sport.

## AUTHOR CONTRIBUTIONS

**Christophe Jacquet**: Data curation; writing—review and editing. **Antoine Piercecchi**: Data curation; writing—review and editing. **Tomas Pineda**: Formal analysis; writing—original draft. **Nicolás Gaggero**: Formal analysis; writing—original draft. **Kristian Kley**: Supervision; writing—review and editing. **Matthieu Ollivier**: Supervision; methodology.

## CONFLICT OF INTEREST STATEMENT

Matthieu Ollivier is a paid consultant and receives royalties from Newclip and Stryker. Kristian Kley is a paid consultant and receives royalties from Newclip. The remaining authors declare no conflict of interest.

## ETHICS STATEMENT

Institutional review board approval was obtained from Aix‐Marseille University, N° Cse_pads25_032_dgr. Informed consent for research use of the tissues was obtained from all donors or their legal representatives.

## Data Availability

The data sets generated and analysed during the current study are available from the corresponding author upon reasonable request.

## References

[1] Caubère A , Barbier O , Kley K , Hanak L , Jacquet C , Ollivier M . Double level osteotomy for genu varum: is a return to sport possible? Orthop Traumatol Surg Res. 2023;109(4):103397. 36087834 10.1016/j.otsr.2022.103397

[2] Dawson M , Elson D , Claes S , Predescu V , Khakha R , Espejo‐Reina A , et al. Osteotomy around the painful degenerative varus knee has broader indications than conventionally described but must follow a strict planning process: ESSKA Formal Consensus Part I. Knee Surg Sports Traumatol Arthrosc. 2024;32(7):1891–1901. 38738832 10.1002/ksa.12256

[3] Guarino A , Farinelli L , Iacono V , Cozzolino A , Natali S , Zorzi C , et al. Long‐term survival and predictors of failure of opening wedge high tibial osteotomy. Orthop Surg. 2023;15(4):1002–1007. 36782306 10.1111/os.13674 PMC10102285

[4] Herbst M , Schröter S , Ateschrang A , Ihle C , Finger F , Histing T , et al. Mid‐term survival and physiological joint angles after double level osteotomy of severe varus osteoarthritis. Knee Surg Sports Traumatol Arthrosc. 2025;33(11):3963–3974. 40652372 10.1002/ksa.12754 PMC12582231

[5] Hoorntje A , Pronk Y , Brinkman JM , van Geenen RCI , van Heerwaarden RJ . High tibial osteotomy versus unicompartmental knee arthroplasty for Kellgren–Lawrence grade 3–4 knee osteoarthritis in younger patients: comparable improvements in patient‐reported outcomes, adjusted for osteoarthritis grade and sex. Knee Surg Sports Traumatol Arthrosc. 2023;31(11):4861–4870. 37572139 10.1007/s00167-023-07526-5 PMC10598142

[6] Jacquet C , Gulagaci F , Schmidt A , Pendse A , Parratte S , Argenson J‐N , et al. Opening wedge high tibial osteotomy allows better outcomes than unicompartmental knee arthroplasty in patients expecting to return to impact sports. Knee Surg Sports Traumatol Arthrosc. 2020;28(12):3849–3857. 32008058 10.1007/s00167-020-05857-1

[7] Jin C , Song E‐K , Santoso A , Ingale PS , Choi I‐S , Seon J‐K . Survival and risk factor analysis of medial open wedge high tibial osteotomy for unicompartment knee osteoarthritis. Arthroscopy. 2020;36(2):535–543. 31901391 10.1016/j.arthro.2019.08.040

[8] Longo UG , Papalia R , Campi S , De Salvatore S , Piergentili I , Bandini B , et al. Evaluating the minimum clinically important difference and patient acceptable symptom state for the WOMAC osteoarthritis index after unicompartmental knee arthroplasty. J Clin Med. 2023;12(24):7618. 38137685 10.3390/jcm12247618 PMC10744230

[9] Lott A , James MG , Kaarre J , Höger S , Kayaalp ME , Ollivier M , et al. Around‐the‐knee osteotomies part II: surgical indications, techniques and outcomes—state of the art. J ISAKOS. 2024;9(4):658–671. 38604568 10.1016/j.jisako.2024.04.002

[10] Mabrouk A , Risebury M , Mumith A , Yasen S . Medial opening wedge high tibial osteotomy yields comparable outcome across all Kellgren–Lawrence osteoarthritis grades. Knee Surg Sports Traumatol Arthrosc. 2025. 10.1002/ksa.70100 41144725

[11] Micicoi G , Ollivier M , Bouguennec N , Batailler C , Tardy N , Rochcongar G , et al. Osteotomies for genu varum: Should we always correct at the tibia? A multicenter analysis of practices in France. Orthop Traumatol Surg Res. 2025;111(1):103925. 38964499 10.1016/j.otsr.2024.103925

[12] Mohammad HR , Judge A , Dodd C , Murray D . The effect of activity on the outcome of cementless mobile bearing unicompartmental knee replacements. Knee. 2023;42:153–160. 37003091 10.1016/j.knee.2023.03.005

[13] Noble PC , Gordon MJ , Weiss JM , Reddix RN , Conditt MA , Mathis KB . Does total knee replacement restore normal knee function? Clin Orthop Relat Res. 2005;431:157–165. 10.1097/01.blo.0000150130.03519.fb 15685070

[14] Ollivier B , Berger P , Depuydt C , Vandenneucker H . Good long‐term survival and patient‐reported outcomes after high tibial osteotomy for medial compartment osteoarthritis. Knee Surg Sports Traumatol Arthrosc. 2021;29(11):3569–3584. 32909057 10.1007/s00167-020-06262-4

[15] Ollivier M , Kley K , Pareek A , Parratte S , Hirschmann MT . Critical considerations in the selection between knee osteotomy and unicompartmental knee arthroplasty in younger patients with varus alignment and early‐stage knee osteoarthritis. Knee Surg Sports Traumatol Arthrosc. 2025;33(10):3445–3453. 40679265 10.1002/ksa.12801

[16] Onishi S , Jacquet C , Nakayama H , Argenson JN , Ollivier M . Opening wedge high tibial osteotomy yields comparable to superior outcomes to unicompartmental knee arthroplasty at 2 years of follow‐up in patients suffering from Ahlbäck III knee osteoarthritis: a propensity score‐matched analysis. J Exp Orthop. 2024;11(4):e70105. 39624640 10.1002/jeo2.70105 PMC11609990

[17] Pratobevera A , Seil R , Menetrey J . Joint line and knee osteotomy. EFORT Open Rev. 2024;9(5):375–386. 38726996 10.1530/EOR-24-0037 PMC11099584

[18] Sohn S , Koh IJ , Kim MS , Kang BM , In Y . What factors predict patient dissatisfaction after contemporary medial opening‐wedge high tibial osteotomy? J Arthroplasty. 2020;35(2):318–324. 31630965 10.1016/j.arth.2019.09.026

[19] Teo SJ , Purnomo G , Koh DTS , Soong J , Yeo W , Razak HRBA , et al. High tibial osteotomy versus unicompartmental knee arthroplasty in advanced medial compartmental knee arthrosis: a comparative study with propensity score matched analysis. Knee. 2024;49:116–124. 38909589 10.1016/j.knee.2024.06.003

[20] Walker LC , Clement ND , Bardgett M , Weir D , Holland J , Gerrand C , et al. The WOMAC score can be reliably used to classify patient satisfaction after total knee arthroplasty. Knee Surg Sports Traumatol Arthrosc. 2018;26(11):3333–3341. 29484445 10.1007/s00167-018-4879-5

[21] Waseem MH , Abideen Zul , Khan MH , Tahir MF , Mukhlis M , Kakakhail A , et al. Comparison of unicompartmental knee arthroplasty versus high tibial osteotomy for medial knee osteoarthritis: an updated meta‐analysis of 56,000 patients. Orthopaedic Surgery. 2025;17(9):2499–2513. 40694375 10.1111/os.70049 PMC12404872

[22] Weiss JM , Noble PC , Conditt MA , Kohl HW , Roberts S , Cook KF , et al. What functional activities are important to patients with knee replacements? Clin Orthop Relat Res. 2002;404:172–188. 10.1097/00003086-200211000-00030 12439258

